# Ferroelectric Polarization Enhanced Photodetector Based on Layered NbOCl_2_

**DOI:** 10.1002/smsc.202300246

**Published:** 2024-01-06

**Authors:** Muyang Huang, Siwei Luo, Hui Qiao, Bowen Yao, Zongyu Huang, Ziyu Wang, Qiaoliang Bao, Xiang Qi

**Affiliations:** ^1^ Hunan Key Laboratory for Micro-Nano Energy Materials and Devices School of Physics and Optoelectronic Xiangtan University Hunan 411105 China; ^2^ Suzhou Institute of Wuhan University Suzhou 215125 China; ^3^ Institute of Energy Materials Science (IEMS) University of Shanghai for Science and Technology Shanghai 200093 China

**Keywords:** ferroelectricity, few-layer, NbOCl_2_, photodetectors

## Abstract

NbOCl_2_ is an emerging ferroelectric layered material with unique optoelectronic properties, in which the built‐in electric field caused by spontaneous polarization can independently drive the separation and transport of photoexcited electrons and holes. However, the optoelectronic performance of NbOCl_2_ and its device application have remained elusive. Here, few‐layer NbOCl_2_ is prepared by the liquid exfoliation method and used to construct photoelectrochemical (PEC)‐type photodetectors. The photodetectors are self‐powered with broadband photoresponse and long‐term cycle stability. Due to the built‐in electric field generated by the spontaneous polarization, the whole system exhibits an open circuit potential of approximately 0.205 V. Interestingly, the open circuit potential can be significantly increased to 0.446 V after poling treatment. The responsivity without external bias is increased by about 2.5 times after 1 V poling and by about 4 times after a poling time of 500 s. Moreover, the tunable ferroelectric polarization shows memory effect and retains about 25% enhancement in photocurrent density even after 60 min. The tuneability of the built‐in electric field in PEC systems based on NbOCl_2_ offers numerous possibilities for the development of photodetectors and nonvolatile memory devices.

## Graphical abstract

The emerging layered ferroelectric NbOCl_2_ is used to construct photoelectrochemical (PEC)‐type photodetectors. Ferroelectric polarization can significantly improve the optoelectronic performance. The tunable ferroelectric polarization shows interesting memory effect. The tuneability of built‐in electric field in PEC systems offers numerous possibilities for the development of optoelectronic devices.

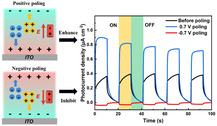

## 1 Introduction

Layered semiconductor materials with suitable bandgap, high carrier mobility, and anisotropy have attracted widespread research interest due to their enormous application potential in optoelectronics,^[^ ^1^, ^2^, ^3^, ^4^, ^5^ ^]^ catalysis,^[^ ^6^, ^7^, ^8^, ^9^ ^]^ energy storage,^[^ ^10^, ^11^, ^12^ ^]^ and communication.^[^ ^13^ ^]^ However, most layered semiconductors require external driving forces to separate and transport photoexcited charge carriers, which limit their potential for self‐driven or low‐energy consumption devices. Fortunately, ferroelectric materials have unique ferroelectric–photon interaction, which are able to independently drive the separation and transport of photoexcited carriers, and have been proven to show significant advantages in photodetector applications.^[^ ^14^, ^15^, ^16^, ^17^, ^18^ ^]^ For example, Wang et al. fabricated a ferroelectric polymer film gated triple‐layer MoS_2_ in which stable remnant polarization helps to provide improved detector sensitivity.^[^ ^19^ ^]^ Wu et al. reported that the carrier type can be modulated by the polarization of ferroelectric polymers deposited on 2D transition metal dihalides. This method had also been used to create a quasi‐nonvolatile memory with a refresh time of 100 s and a write/erase speed of 10 μs.^[^ ^20^ ^]^ Zhao et al. demonstrated a novel photovoltaic–pyroelectric system. When compared to a purely photovoltaic system, the corresponding current peak and plateau in the coupled photovoltaic–pyroelectric system were 451.9% and 17.2% higher, respectively.^[^ ^21^ ^]^ These results underscore that the interaction between ferroelectric domains, electrons, and photons is one of the effective strategies to modulate the optoelectronic properties, and plays an important role for the improvement of optoelectronic performance.

Most recently, NbOCl_2_, one of the niobium oxide dihalides NbOX_2_ (X = Cl, Br, I) family, has attracted great attention as an emerging ferroelectric layered material.^[^ ^22^, ^23^ ^]^ Guo et al. reported that NbOCl_2_ has a layer decoupled bandgap (2.1 eV), monolayer‐like excitonic behavior in the bulk form, along with a scalable second‐harmonic generation intensity. The spontaneous parametric down‐conversion process observed in NbOCl_2_ ultrathin van der Waals flakes makes it promising for applications in quantum light sources and sensors.^[^ ^24^ ^]^ Abdelwahab et al. reported that few‐layer NbOCl_2_ have strong nonlinear optical effects. An upconversion efficiency of up to 0.004% provides the possibility for designing optical cross‐correlators for ultrashort pulses characterization.^[^ ^25^ ^]^ In addition, Su et al. reported that NbOCl_2_ has high carrier mobility (≈10^4^ cm^2^ V^−1^ s^−1^) and strong light absorption in the visible range, implying great application potential in optoelectronics.^[^ ^26^ ^]^ More importantly, the positive and negative charge centers of NbOCl_2_ do not coincide, causing spontaneous polarization and generating a built‐in electric field. This built‐in electronic field can independently drive the separation and transmission of photoexcited carriers.^[^ ^27^, ^28^, ^29^ ^]^ Interestingly, the direction and intensity of the built‐in electric field have been proven to be modulated by the external electric field,^[^ ^18^, ^19^ ^]^ implying that it is feasible to improve the optoelectronic performance by adjusting the ferroelectric polarization. It is known that the control of carrier behavior is crucial for optoelectronic performance,^[^ ^30^, ^31^ ^]^ but this has not been observed in ferroelectric NbOCl_2_.

In this work, we report ferroelectric polarization enhanced PEC‐type photodetectors based on layered NbOCl_2_. The NbOCl_2_‐based photodetectors are self‐powered with broadband photoresponse and long‐term cycle stability. The ferroelectric polarization of NbOCl_2_ electrode was modulated by poling treatment, and the open circuit potential can be increased significantly after positive poling. At the same time, the photocurrent density increased several times without external bias. More interestingly, the photocurrent density remains significantly higher than that of unpolarized even after a long time. We provide a feasible strategy for PEC systems based on NbOCl_2_ or other ferroelectric materials, which paves a new way for developing novel optoelectronic devices.

## 2 Results and Discussion

**Figure 1:**
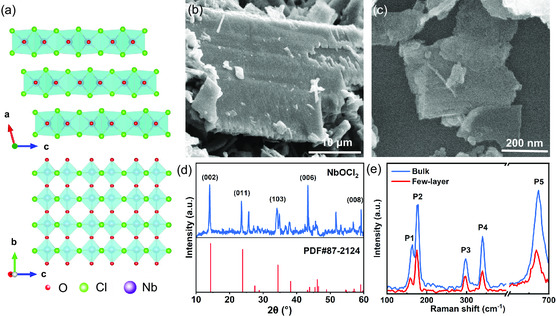
a) Schematic diagram of the atomic structure model of NbOCl_2_. The red, green, and purple spheres represent oxygen, chlorine and niobium atoms, respectively. b) SEM image of bulk NbOCl_2_. c) SEM image of few‐layer NbOCl_2_. d) XRD patterns of NbOCl_2_ and its standard card. e) Raman spectrum of NbOCl_2_ bulk and few‐layer.

The atomic structure diagram of NbOCl_2_ is shown in **Figure** **1**a. It belongs to C_2_ space group and has van der Waals stacking behavior. Interestingly, the location of the Nb atom is not in the center of the octahedron, which makes NbOCl_2_ have spontaneous polarization, laying the foundation for the construction of self‐powered photodetectors. According to previous reports, NbOCl_2_ crystals have the opportunity to obtain few‐layers.^[^ ^24^, ^25^ ^]^ Here, the few‐layer NbOCl_2_ were obtained through the liquid exfoliation method. Figure S1, Supporting Information, (left) shows a schematic diagram of the typical preparation method for few‐layer NbOCl_2_ based on liquid exfoliation method. The detailed preparation process is described in the experimental section. The microstructure of few‐layer NbOCl_2_ was characterized by scanning electron microscopy (SEM). Figure 1b,c show the SEM images of NbOCl_2_ bulk and few‐layers, respectively. It can be seen that the obtained few‐layer NbOCl_2_ still have a complete layered structure. In addition, Raman spectrum and X‐ray diffraction (XRD) were used to characterize the crystal structure. Figure 1d shows the XRD spectrum of NbOCl_2_, which matches well with the standard PDF card (#87‐2124) of NbOCl_2_ crystal. As shown in Figure S2a, Supporting Information, more diffraction peaks may come from water (PDF card #85‐0800) because chlorides are more likely to absorb water. Figure 1e shows the Raman spectrum of NbOCl_2_ bulk and few‐layer. Among them, the Raman peaks located at 160, 179, 298, 340, 670 cm^−1^ are assigned to P1, P2, P3, P4, and P5 modes, respectively. Compared to bulk NbOCl_2_, the Raman peaks of few‐layer NbOCl_2_ do not show significant changes. Photoluminescence (PL) spectrum was used to evaluate the bandgap of few‐layer NbOCl_2_. Figure S2b, Supporting Information, shows the PL spectrum of few‐layer NbOCl_2_. The PL peak is located near 1.95 eV, which is consistent with the previous reports.^[^ ^24^ ^]^ Finally, the light absorption property was analyzed using UV‐visible absorption spectroscopy. The absorption spectrum (Figure S2c, Supporting Information) shows a wide absorption region, indicating strong absorption in the visible light region. The successful acquisition of high‐quality few‐layer NbOCl_2_ lays the foundation for the next step of PEC‐based optoelectronic testing.

**Figure 2:**
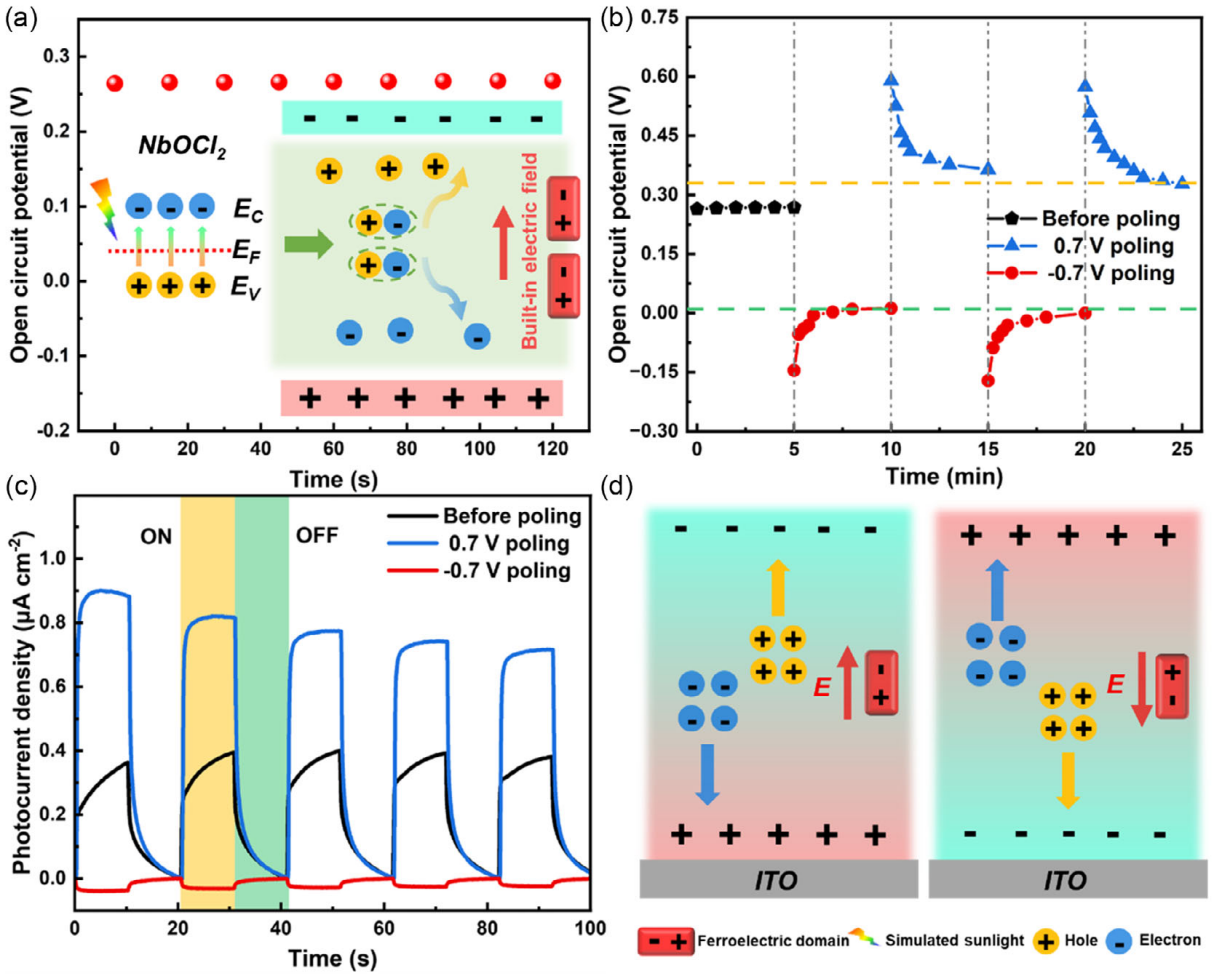
a) Stable open circuit potential over time and schematic diagram of built‐in electric field in PEC system. b) Open circuit potential changes after ±0.7 V poling. c) Photocurrent density curves at open circuit potential after poling. d) Schematic diagram of photoexcited carrier separation and transmission driven by built‐in electric field.

Optoelectronic performance test was conducted to evaluate the photoresponse performance of few‐layer NbOCl_2_ under simulated sunlight. Figure S1, Supporting Information, (right) shows the typical PEC‐type testing system. The 0.5 m Na_2_SO_4_ solution was used as the electrolyte for the entire measurement system. Firstly, we focused on the open circuit potential before poling. As shown in **Figure** **2**a, it can be seen that the open circuit potential is stable around 0.26 V. Generally, the open circuit potential in the PEC system is the potential difference between the working electrode and the reference electrode. The open circuit potential of the whole system is composed of several parts, mainly including the built‐in electric field of the material on the indium tin oxide (ITO) substrate and the interface barrier between the material and the electrolyte. For electrolytes, poling treatment (see the experimental section) does not affect the barrier height. However, the poling treatment will cause the built‐in electric field to change, so the interface barrier between the material and the electrolyte will also change, which will inevitably lead to changes in the whole system. Therefore, the open circuit potential is used to reflect the built‐in electric field intensity in the NbOCl_2_ electrode. It should be noted that when the bias of the electrochemical workstation is set to open circuit potential, the electrochemical workstation actually does not output bias, which is used to obtain the photoresponse without external bias. In the following discussion, the bias is set to the open circuit potential unless otherwise specified. As shown in Figure S3, Supporting Information, the working electrode exhibits obvious “on/off” behavior at open circuit potential, and the photocurrent density is about 370 nA cm^−2^. This indicates that the photodetector exhibits self‐powered behavior, and the built‐in electric field generated by spontaneous polarization plays an important role in the driving and transmission of photoexcited carriers. As shown in the illustration in Figure 2a, the ferroelectric domains inside the material have a built‐in electric field, and photoexcited electrons and holes are separated and transported under the drive of the built‐in electric field. Next, we attempt to modulate the direction and intensity of spontaneous polarization by poling treatment (see the experimental section). As shown in Figure 2b, after negative (positive) poling, the open circuit potential tends to stabilize around 0.01 V (0.34 V). For the sake of comparison, the 200 s poling process is omitted in the Figure 2b. It can be seen that the open circuit potential shows obvious modulation behavior. This indicates that poling treatment reconstructed the polarization direction and intensity of the electrode. Next, the photoresponse of the photodetector was tested without external bias, as shown in Figure 2c. It can be seen that the electrode before poling exhibits anodic photocurrent, and the photocurrent significantly increases after 0.7 V poling, indicating that the polarization intensity of the NbOCl_2_ electrode is further enhanced. However, after −0.7 V poling, the electrode exhibits cathode photocurrent under illumination. Figure 2d shows the schematic diagram of the charge distribution of NbOCl_2_ at different polarization states. After poling, if the direction of ferroelectric polarization is the same as the external electric field, the polarization intensity increases; otherwise, the polarization intensity weakens or even reverses. The results show that the poling treatment can effectively adjust the polarization direction and intensity of NbOCl_2_ electrode, which makes it obtain the ability to switch between the anode and the cathode electrode, and significantly expand the application range.

**Figure 3:**
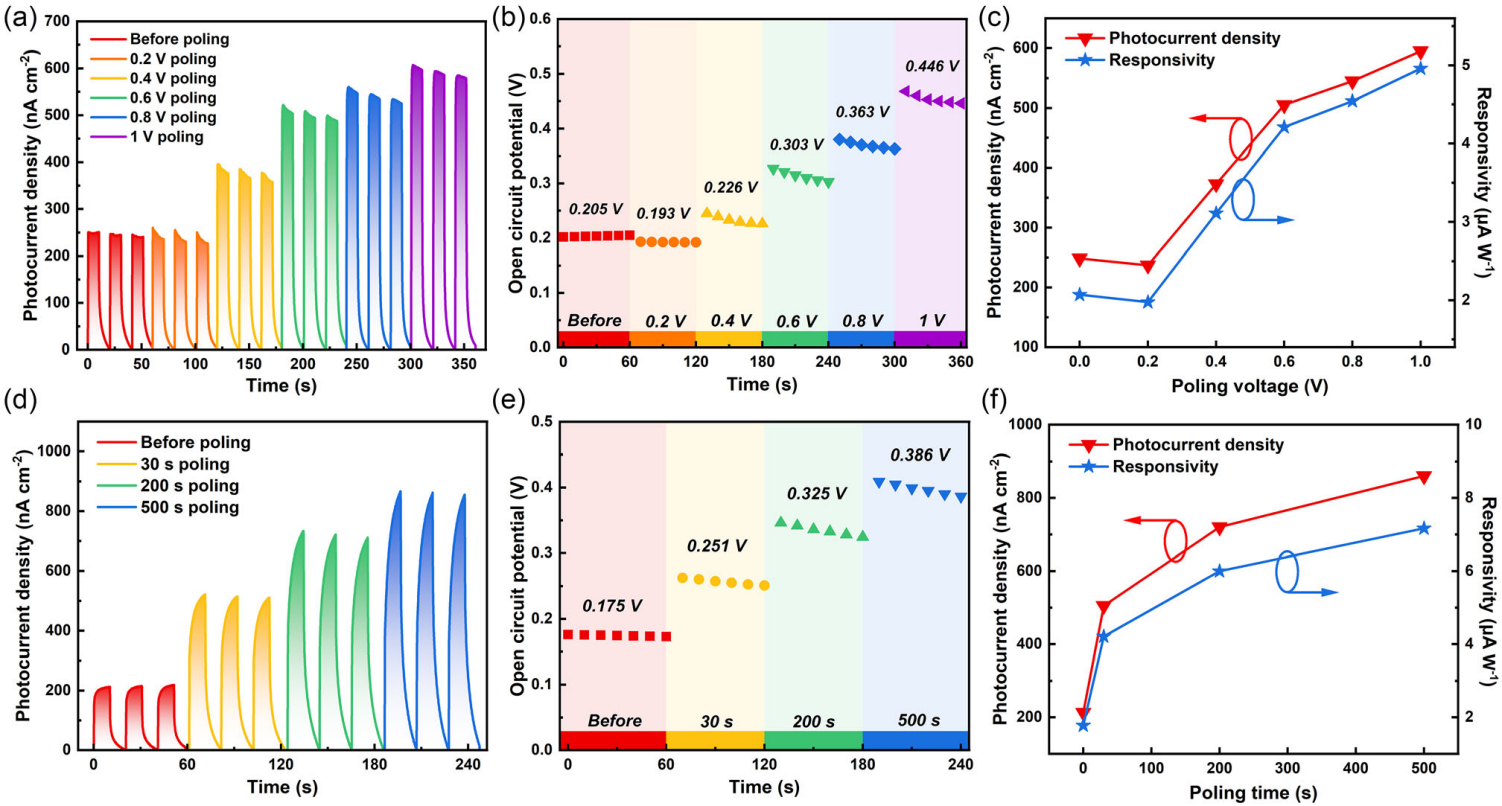
a) Photocurrent density curves without external bias after poling treatment with different poling biases. b) Open circuit potential after poling treatment corresponding to (a). c) Photocurrent density and responsivity after poling treatment corresponding to (a). d) Photocurrent density curves without external bias after poling treatment with different poling time. e) Open circuit potential after poling treatment corresponding to (d). f) Photocurrent density and responsivity after poling treatment corresponding to (d).

In order to further investigate the effect of ferroelectric polarization on optoelectronic performance, the electrodes were treated with different poling bias for 200 s. The photocurrent density curves obtained without external bias after poling is shown in **Figure** **3**a. It can be seen that the photocurrent density increases from 248 nA cm^−2^ before poling to 595 nA cm^−2^ after 1 V poling. This indicates that poling treatment can effectively enhance the optoelectronic performance of NbOCl_2_ electrode. In addition, when the poling bias is higher than 0.8 V, the growth rate of the photocurrent density is reduced, which may be due to slight damage to the sample at high bias. It is worth noting that after 0.2 V poling, the photocurrent density shows a slight decrease, which is attributed to the poling bias lower than the open circuit potential. The open circuit potential after poling corresponding to Figure 3a is shown in Figure 3b. The open circuit potential of the electrode before poling is about 0.205 V, and it actually decreases after 0.2 V poling. This is due to the fact that the actual output bias of the electrochemical workstation during the electrochemical processing was −0.005 V. Subsequently, with the increase of poling bias, the open circuit potential increases steadily. The synchronous increase in photocurrent density is attributed to a higher open circuit potential can drive the separation and transport of photoexcited electrons and holes more effectively. In addition, responsivity (*R*) is introduced to evaluate the photoresponse under different polarization intensities. The *R* is obtained by the formula *R* = *I*/*J*_light_,^[^ ^32^ ^]^ where I is the photocurrent density and *J*_light_ is the light irradiation intensity (120 mW). The photocurrent density and responsivity without external bias are shown in Figure 3c. It can be seen that the responsivity increases with the increase of poling bias, from 2.071 to 4.958 μA W^−1^. The specific data above is shown in Table S1, Supporting Information. Similar to positive polarization, negative poling also has a significant effect on the performance of photodetectors. As shown in Figure S4a, Supporting Information, after negative poling, the photocurrent density without external bias decreases significantly, which is attributed to the application of an electric field in the opposite direction during negative poling. The decrease of electrode polarization intensity leads to the decrease of open circuit potential, which affects the photocurrent density. In addition, as shown in Figure S4b, Supporting Information, the working electrode change from anode photocurrent to cathode photocurrent after negative poling, which can be attributed to the reversal of ferroelectric polarization direction under negative poling. However, it should be noted that the cathode photocurrent behavior is not stable, which may be due to the higher poling bias required to achieve an open circuit potential of less than 0 V. Finally, typical electrochemical workstation settings were used to test the photoresponse at different bias, as shown in Figure S5a,b, Supporting Information. The typical electrochemical workstation setting refers to setting the bias directly to 0 V. The positive bias or negative bias is selected based on the direction of photocurrent at 0 V. Here, the optoelectronic performance was tested under bias of −0.2, −0.4, −0.6, and −0.8 V, respectively. More details are provided in the supporting information.

For ferroelectric domains, the enhancement or reversal of ferroelectric polarization usually requires sufficient poling time. Therefore, the effect of different poling times on the polarization intensity of the electrode is discussed as follows. Here, the electrodes were treated by 0.7 V poling bias for 30, 200, and 500 s, respectively. Figure 3d shows the photocurrent density curves without external bias after poling for different times. It can be seen that the photocurrent density increases significantly with the increase of poling time. The photocurrent density after a poling time of 500 s is about 4 times that before poling. Figure 3e shows the open circuit potential after different poling times. It can be seen that the open circuit potential after poling is significantly increased, and the longer the poling time, the higher the open circuit potential. Therefore, the appropriate extension of electrochemical processing time is more conducive to the full polarization of NbOCl_2_ electrode. The responsivity after different poling times can be obtained from Figure 3f, where it can be seen that the responsivity increases from 1.775 to 7.167 μA W^−1^. Table S2, Supporting Information, shows the specific data after different poling times. It is worth noting that the responsivity after a poling time of 500 s has a limited improvement compared with the responsivity after a poling time of 200 s, but the poling time will be significantly increased. Therefore, 200 s may be the ideal poling time, which can avoid sample damage after a long poling time while obtaining good performance improvement.

In addition, light irradiation intensity, incident light wavelength, and electrolyte concentration are also important factors affecting the performance of photodetectors. As shown in Figure S6a,b, Supporting Information, the photocurrent density increases linearly with the increase of light irradiation intensity. As shown in Figure S7, Supporting Information, it was found in the wavelength dependence test that the NbOCl_2_‐based photodetector have good photoresponse in the visible light range, and the photocurrent density gradually increases as the wavelength decreases. For different electrolyte environments, the photodetector exhibits improved performance in high concentration electrolytes, as shown in Figure S8a,b, Supporting Information. The results show that the NbOCl_2_‐based photodetector has good adjustability and is one of the strong competitors of PEC‐type photodetector. More details are provided in the supporting information.

**Figure 4:**
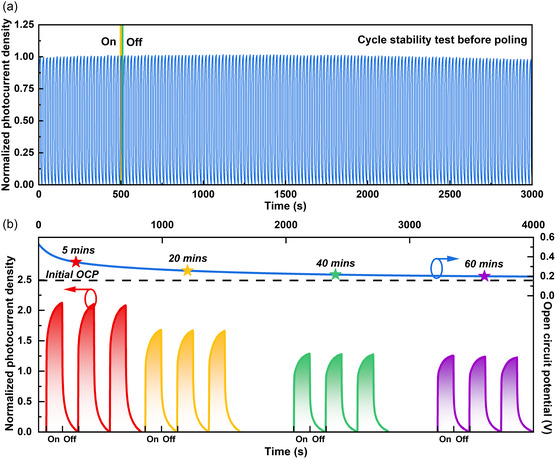
a) Cyclic stability test and normalized photocurrent density curve without external bias before poling. b) Time stability test of the open circuit potential and normalized photocurrent density after poling. The open circuit potential gradually decreases over time and stabilizes after 40 min. There is still a 25% increase in photocurrent density after 60 min.

For ferroelectric materials, the remnant polarization usually decreases over time after the removal of the external electric field, as some ferroelectric domains that are not fully polarized tend to revert to their initial state. Therefore, unlike traditional PEC‐type photodetectors, both the cycle stability and remnant polarization stability affect the performance of ferroelectric photodetectors. Here, the NbOCl_2_ electrode was polarized at 0.7 V poling bias for 200 s, and the photoresponse was tested after 5, 20, 40, and 60 min. To facilitate comparison, the photocurrent density is normalized. As shown in **Figure** **4**a, there is no significant decrease in photocurrent density before poling during the 3000 s test. This proves that NbOCl_2_ photodetector have great cycle stability. Next, we focus on the stability of the polarization state after poling. Figure 4b shows the time‐dependent open circuit potential curve and the photocurrent density curves after 5, 20, 40, and 60 min respectively. The open circuit potential gradually decreased after removing the external electric field, which is due to the fact that partially incomplete inverted ferroelectric domains tend to recover their initial state. The photocurrent density curves at different times can more intuitively reflect the trend of optoelectronic performance. It can be seen that the photocurrent density after 5 min is about 2.2 times that of the pristine sample. The photocurrent density is still about 25% higher than unpolarized even after 60 min. The results show that poling treatment can effectively enhance the optoelectronic performance of NbOCl_2_ electrode, and it still has enhanced optoelectronic performance after removing the external electric field.

## 3 Conclusion

An approach of using ferroelectric polarization to improve and modulate the performance of PEC photodetectors based on layered NbOCl_2_ is demonstrated. Due to the built‐in electric field generated by ferroelectric polarization, the photodetector can operate without external bias. Interestingly, the optoelectronic performance can be effectively tuned by poling treatment. The open circuit potential increases from 0.205 to 0.446 V after poling treatment. The responsivity without external bias is increased by about 2.5 times after 1 V poling and by about 4 times after a poling time of 500 s. In addition, the improved photodetector performance has a memory effect and remains for 60 min after removing the external electric field. The demonstration of tunable built‐in electric field in PEC systems based on ferroelectric materials may offer new opportunities for the development of low‐energy consumption photodetectors and nonvolatile memory devices.

## 4 Experimental Section

4.1

4.1.1

##### Materials

Absolute ethanol (99.7%) and acetone (99.5%) were obtained from Hunan Huihong Reagent Co., Ltd. *N*‐methyl‐2‐pyrrolidone (NMP, 99%) was obtained from Tianjin Kemiou Chemical Reagent Co., Ltd. Bulk NbOCl_2_ was obtained from Nanjing MKNANO Tech. Co., Ltd. The rest of the reagents can be used directly without further purification.

##### Preparation of Few‐Layer NbOCl_2_

The traditional liquid exfoliation method was used to prepare the few‐layer NbOCl_2_. According to previous reports, reducing the layer number of layered materials has been shown to play an important role in improving optoelectronic performance, such as increasing the specific surface area and light absorption efficiency.^[^ ^33^, ^34^, ^35^, ^36^, ^37^ ^]^ Figure S9a,b, Supporting Information, shows the atomic force microscopy image of the NbOCl_2_ flakes, and the photocurrent density of few‐layer and bulk, respectively. The bulk NbOCl_2_ was ground for 30 min to obtain small particles. Then, a certain of bulk NbOCl_2_ was added to a 100 mL beaker with NMP and ultrasonicated at room temperature for 6 h. The dispersion after liquid exfoliation was centrifuged to remove incompletely exfoliated NbOCl_2_ and obtain few‐layer NbOCl_2_ powder. Finally, acetone, ethanol, and deionized water were used to wash the powder twice and then it was freeze‐dried for 12 h. The dried powder was used for the preparation of electrodes.

##### Characterizations and Optoelectronic Performance Test

The micromorphology and structure of few‐layer NbOCl_2_ were characterized using SEM (Hitachi s4800). The Raman spectrum of few‐layer NbOCl_2_ were collected using a Raman microscope (WItec Alpha 300r) with 532 nm laser wavelength at room temperature. The UV–Vis absorption spectrum was measured by UV–Vis Spectrophotometer (UV‐2600i, Shimadzu) in the range of 300–850 nm. The optoelectronic performance test was implemented relying on a standard electrochemical workstation (CHI760E, CH Instruments, Inc.). An ITO glass electrode coated with few‐layer NbOCl_2_ was used as the working electrode, a platinum flake was used as the counter electrode, and an Ag/AgCl electrode was used as the reference electrode. The irradiation intensity of simulated sunlight set to 120 mW cm^−2^. The 0.5 m NaSO_4_ solution was used as electrolytes during the test. Poling treatment refers to the process of polarizing the working electrode by applying a bias using an electrochemical workstation. The default poling bias is 0.7 V and the poling time is 200 s. Here, the maximum poling bias is limited to 1 V because the working electrode in the PEC environment may participate in a chemical reaction at a high bias. The photocurrent density obtained in the test was obtained after removing the external electric field for about 5 min. This is to avoid the unstable stage of the open circuit potential to obtain a stable photocurrent.

## Conflict of Interest

The authors declare no conflict of interest.

## Supporting information

Supplementary Material

## Data Availability

The data that support the findings of this study are available from the corresponding author upon reasonable request.
