# Supplementary material for: Ferroelectric Polarization Enhanced Photodetector Based on Layered NbOCl2
Source: Small Sci. 2024 Jan 6;4(3):2300246. doi: 10.1002/smsc.202300246 (PMC11935227; doi:10.1002/smsc.202300246)
Supplement: Supplementary file 1 — Supplementary Material [file SMSC-4-2300246-s001.pdf]

Ferroelectric Polarization Enhanced Photodetector Based on Layered $NbOCl_2$

Muyang Huang[1], Siwei Luo[1,*], Hui Qiao[1], Bowen Yao[1], Zongyu Huang[1], Ziyu Wang[3], Qiaoliang Bao[2,*], Xiang Qi[1,*]

[1] Hunan Key Laboratory for Micro-Nano Energy Materials and Devices, School of Physics and Optoelectronic, Xiangtan University, Hunan 411105, China

[2] Institute of Energy Materials Science (IEMS), University of Shanghai for Science and Technology, Shanghai 200093, China

[3] Suzhou Institute of Wuhan University, Suzhou 215125, China

[*]Author to whom correspondence should be addressed:
swluo@xtu.edu.cn (Siwei Luo), qiaoliang.bao@usst.edu.cn (Qiaoliang Bao) and xqi@xtu.edu.cn (Xiang Qi)

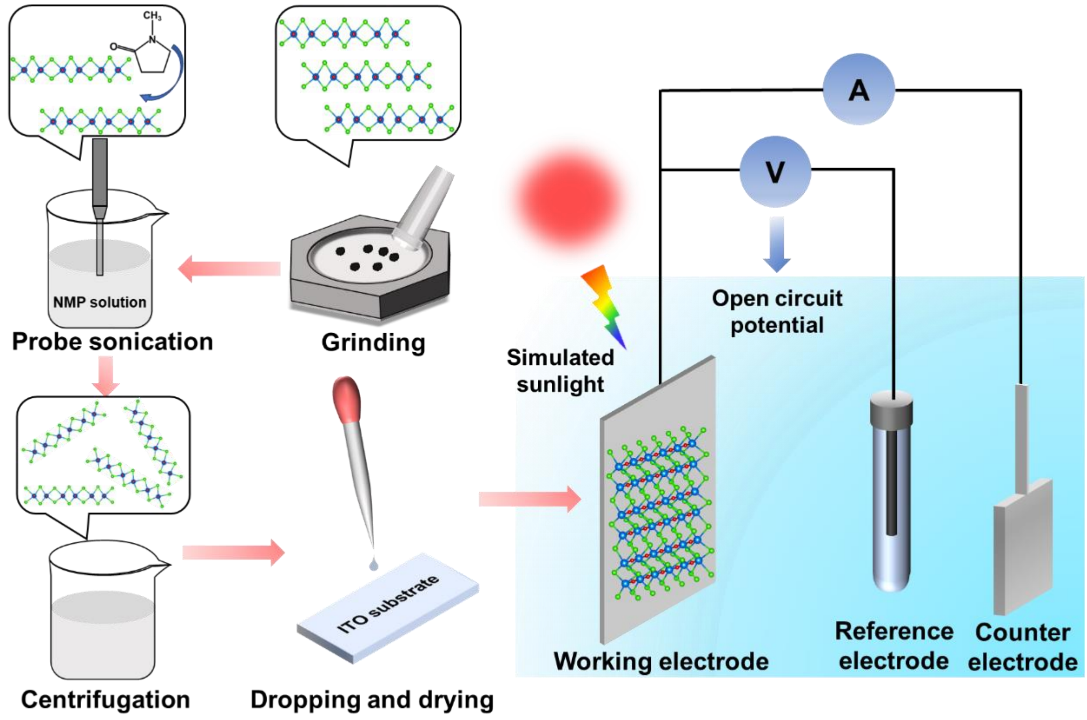

Figure S1 Schematic diagram of preparation method for few-layer $NbOCl_2$ and a typical PEC-type testing system.

The bulk $NbOCl_2$ was ground for 30 min to obtain small particles. Then, a certain of bulk $NbOCl_2$ was added to a 100 ml beaker with NMP and ultrasonicated at room temperature for 6 h. The dispersion after liquid exfoliation was centrifuged to remove incompletely exfoliated $NbOCl_2$ and obtain few-layer $NbOCl_2$ powder. Finally, acetone, ethanol, and deionized water were used to wash the powder twice and then it was freeze-dried for 12 hours. The dried powder was used for the preparation of electrodes. The optoelectronic performance test was implemented relying on a standard PEC-type testing system.

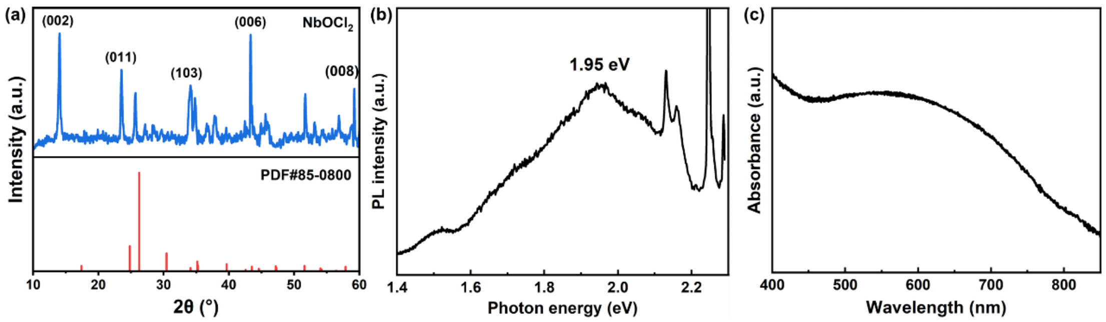

Figure S2 (a) The XRD patterns of $NbOCl_2$ and the standard card of water (PDF card #85-0800). (b) The PL spectrum of few-layer $NbOCl_2$. The band gap is about 1.95 eV. (c) The UV-visible absorbance spectrum of few-layer $NbOCl_2$. The few-layer $NbOCl_2$ shows good absorption in the visible light range.

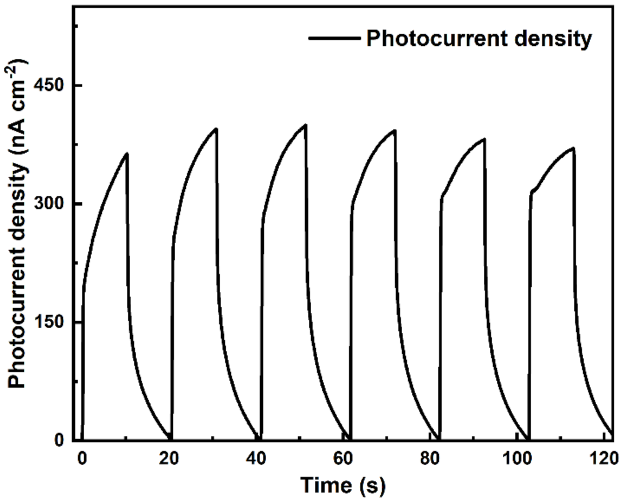

Figure S3 The photocurrent density curves at open circuit potential. The photodetector shows obvious “on/off” switching behavior.

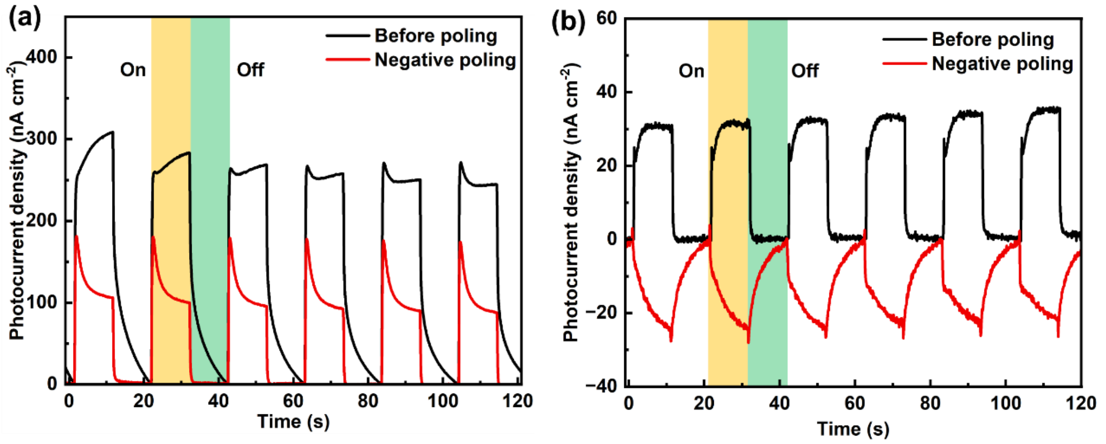

Figure S4 (a, b) The photocurrent density curves at open circuit potential after negative poling.

As shown in Figure S4 (a), after negative poling, the photocurrent density without external bias exhibits a decreasing behavior. As shown in Figure S4 (b), some of the working electrodes after negative poling even show a reversal of the direction of the photocurrent. However, it should be noted that the cathode photocurrent behavior is not stable. Overall, negative poling can significantly inhibit the photocurrent density.

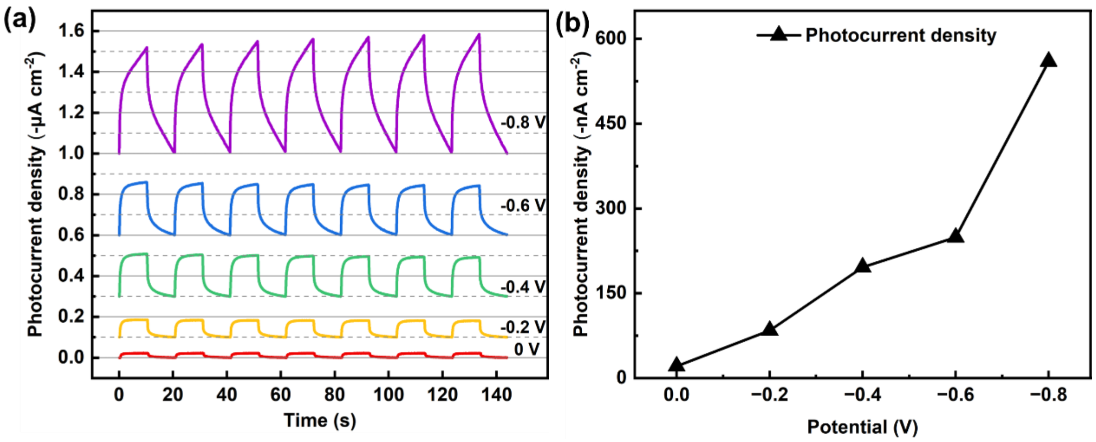

Figure S5 (a, b) The photocurrent density curves at different bias from 0 to -0.8 V.

The "on/off" switching behavior tested at bias of 0 V, -0.2 V, -0.4 V, -0.6 V, and -0.8 V is shown in Figure S5. As the bias increases, the photoresponse gradually improves. The photocurrent increases from -22 nA cm^{-2} at 0 V to -550 nA cm^{-2} at -0.8 V. This is attributed to the fact that high bias is more conducive to electron-hole separation and promotes the movement of the carriers. Especially, when the bias is higher than -0.5 V, the photocurrent density shows a significant improvement. The photocurrent at a bias of -0.8 V is about three times higher than that at a bias of -0.4 V. This may be attributed to the polarization direction reconstruction of ferroelectric $NbOCl_2$.

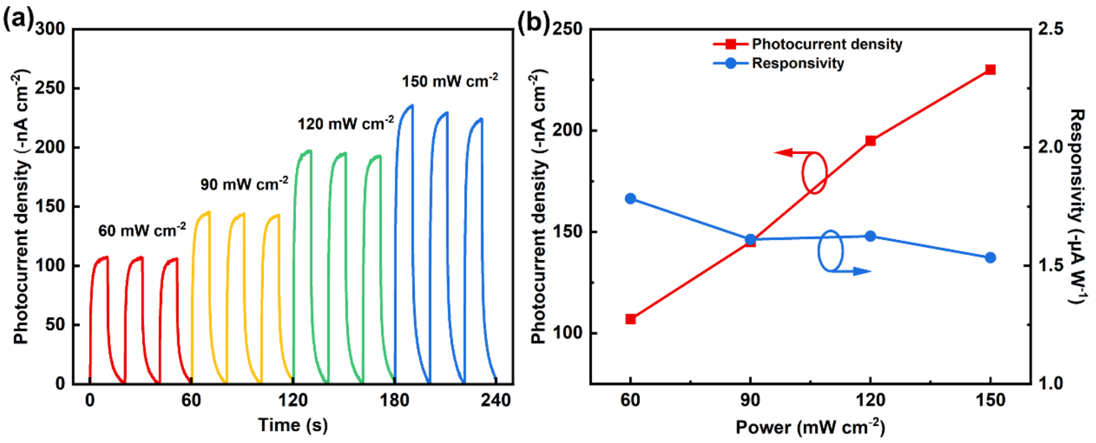

Figure S6 (a) The photocurrent density curves at different light irradiation intensities. (b) The photocurrent density and responsivity at different light irradiation intensities.

The dependence of photocurrent density on light irradiation intensity is another key parameter for evaluating the performance of photodetector. Here, our test was proceeding under a 0.5 M Na_2SO_4 condition and a bias of -0.4 V. As shown in Figure S6 (a), the photocurrent density gradually increases with the increases of light irradiation intensity. The photocurrent density is -235 nA cm^{-2} for 150 mW cm^{-2}, which is two times higher than -107 nA cm^{-2} for 60 mW cm^{-2}. This is attributed to the increase in electrons and holes caused by higher irradiance intensity. As shown in Figure S6 (b), it can be seen that there is a good linear relationship between photocurrent density and light irradiation intensity which is consistent with the expected results. The results show that the responsivity of the electrode varies from -1.53 μA W^{-1} to -1.78 μA W^{-1}.

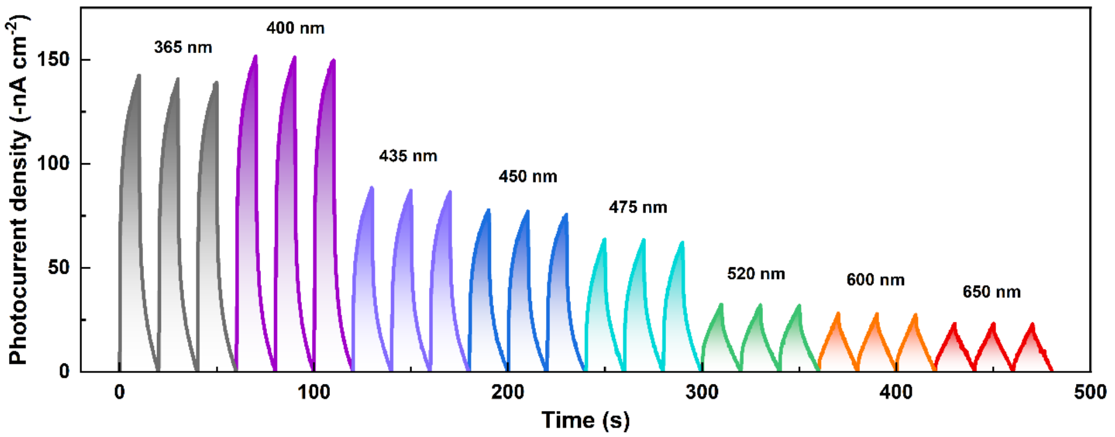

Figure S7 The photocurrent density at different light wavelengths.

The wavelength of light is also an important factor affecting the performance of photodetectors. Filters with different wavelengths are used to adjust the wavelengths of light (365 nm, 400 nm, 435 nm, 450 nm, 475 nm, 520 nm, 600 nm, 650 nm), while the light irradiation intensity is maintained at 120 mW cm^{-2} and the bias was set to -0.4 V. As shown in Figure S7, as the wavelength of light decreases, its photocurrent density gradually increases. This indicates that $NbOCl_2$ based photodetectors may be more suitable for working in short wavelengths. When approaching the ultraviolet band, the photocurrent density reaches saturation and slightly decreases, which could be attributed to its reduced absorption of ultraviolet light.

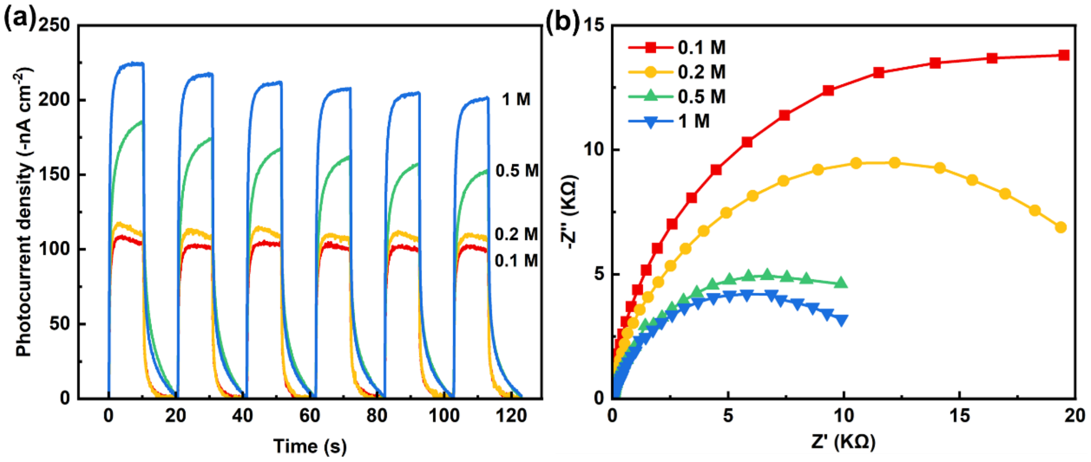

Figure S8 (a) The photocurrent density curves at different concentrations. (b) The EIS map in different Na_2SO_4 electrolyte concentrations.

Electrolyte concentration is also one of the factors that need to be considered for the performance of photodetectors. Here, the photoresponse and electrochemical impedance spectroscopy (EIS) were tested in 0.1 M, 0.2 M, 0.5 M, and 1 M Na_2SO_4 solutions, with bias voltage controlled at -0.4 V and light irradiation intensity maintained at 120 mW cm^{-2}. EIS reflects the resistance at the interface between the electrode and electrolyte, as well as the efficiency of charge transfer. From Figure S8 (a), it can be intuitively seen that the photoresponse is stronger in high concentration electrolyte solutions. The photocurrent density doubles under the condition of 1 M Na_2SO_4 compared to 0.1 M. This is because high concentrations of electrolytes can provide more conducting ions. As shown in Figure S8 (b), as the concentration increases, the curve diameter continuously decreases, indicating that the contact resistance of the $NbOCl_2$ electrode decreases with the increase of electrolyte concentration. Therefore, at high concentrations, the resistance between interfaces decreases, improving charge transfer efficiency and photocurrent density.

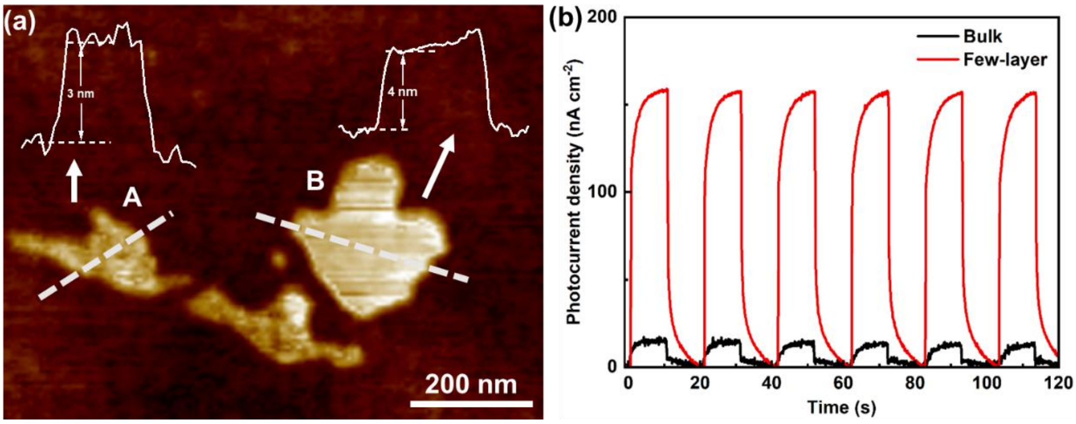

Figure S9 (a) The AFM image of few-layer $NbOCl_2$. (b) Photocurrent density curves of bulk and few-layer $NbOCl_2$.

Figure S9 (a) shows the AFM image of the $NbOCl_2$ flakes. It can be seen that the thickness is about 4 nm, which indicates that we have successfully reduced the thickness. As shown in Figure S9 (b), the photocurrent density of few-layer $NbOCl_2$ is much higher than that of the bulk.

Table S1 Open circuit potential, photocurrent density and responsivity after poling treatment with different poling biases.

Poling bias (V)	Open circuit potential (V)	Photocurrent density (nA cm^{-2})	Responsivity (μA W^{-1})
0	0.205	248	2.071
0.2	0.193	237	1.976
0.4	0.226	373	3.108
0.6	0.303	505	4.208
0.8	0.363	545	4.542
1	0.446	595	4.958

Table S2 Open circuit potential, photocurrent density and responsivity after poling treatment with different poling times.

Poling time (s)	Open circuit potential (V)	Photocurrent density (nA cm^{-2})	Responsivity (μA W^{-1})
0	0.175	213	1.775
30	0.251	505	4.208
200	0.325	720	6.000
500	0.386	860	7.167
